# The transcriptional landscape and biomarker potential of circular RNAs in prostate cancer

**DOI:** 10.1186/s13073-021-01009-3

**Published:** 2022-01-25

**Authors:** Emma Bollmann Hansen, Jacob Fredsøe, Trine Line Hauge Okholm, Benedicte Parm Ulhøi, Søren Klingenberg, Jørgen Bjerggaard Jensen, Jørgen Kjems, Kirsten Bouchelouche, Michael Borre, Christian Kroun Damgaard, Jakob Skou Pedersen, Lasse Sommer Kristensen, Karina Dalsgaard Sørensen

**Affiliations:** 1 Department of Molecular Medicine (MOMA), Aarhus University Hospital, Palle Juul-Jensens Boulevard 99, 8200 Aarhus, Denmark; 2 Department of Clinical Medicine, Aarhus University, Aarhus, Denmark; 3 Department of Pathology, Aarhus University Hospital, Aarhus, Denmark; 4 Department of Nuclear Medicine and PET, Aarhus University Hospital, Aarhus, Denmark; 5 Department of Urology, Regional Hospital of West Jutland, Holstebro, Denmark; 6 Department of Molecular Biology and Genetics (MBG), Aarhus University, Aarhus, Denmark; 7 Interdisciplinary Nanoscience Center (iNANO), Aarhus University, Aarhus, Denmark; 8 Department of Urology, Aarhus University Hospital, Aarhus, Denmark; 9 Bioinformatics Research Centre, Aarhus University, Aarhus, Denmark; 10 Department of Biomedicine, Aarhus University, Aarhus, Denmark

**Keywords:** Prostate, Cancer, Biomarker, circRNA

## Abstract

**Background:**

Circular RNAs (circRNAs) constitute a largely unexplored source for biomarker discovery in prostate cancer (PC). Here, we characterize the biomarker potential of circRNAs in PC, where the need for novel diagnostic and prognostic tools to facilitate more personalized management is pressing.

**Methods:**

We profiled the transcriptomic landscape of circRNAs in PC by total RNA sequencing of 31 adjacent-normal and 143 tumor samples from localized (radical prostatectomy (RP)) and metastatic PC patients (cohort 1, training). Diagnostic and prognostic potential was evaluated in cohort 1, and 39 top circRNA candidates were selected for validation in two additional PC cohorts (cohort 2, *n* = 111; RP cohort 3, *n* = 191) by NanoString-based expression analysis. Biochemical recurrence (BCR)-free survival was assessed using Kaplan-Meier, univariate, and multivariate Cox regression analyses. The circRNA candidates were further detected in extracellular vesicle (EV)-enriched plasma samples from PC patients and controls (cohort 4, *n* = 54).

**Results:**

Expression of circABCC4, circFAT3, circATRNL1, and circITGA7 was highly cancer-specific (area under the curve 0.71–0.86), while low circITGA7 expression was significantly (*P* < 0.05) associated with BCR in univariate analysis in two RP cohorts. Moreover, we successfully trained and validated a novel 5-circRNA prognostic signature (circKMD1A/circTULP4/circZNF532/circSUMF1/circMKLN1) significantly associated with BCR beyond routine clinicopathological variables (RP cohort 1: *P* = 0.02, hazard ratio = 2.1; RP cohort 3: *P* < 0.001, hazard ratio = 2.1). Lastly, we provide proof-of-principle for detection of candidate circRNAs in EV-enriched plasma samples from PC patients.

**Conclusions:**

circRNAs hold great biomarker potential in PC and display both high cancer specificity and association to disease progression.

**Supplementary Information:**

The online version contains supplementary material available at 10.1186/s13073-021-01009-3.

## Background

Prostate cancer (PC) is the second most frequently diagnosed non-cutaneous cancer in men worldwide and the third leading cause of cancer-associated mortality in men in Western Europe and the USA [1].

Whereas localized PC (LPC) is curable by radical prostatectomy (RP), metastatic PC (MPC) is incurable with a 5-year survival rate below 40% [2, 3]. Early diagnosis is thus essential for long-term patient survival. Still, approximately 30% of patients with LPC who undergo curatively intended RP experience relapse within 10 years [4]. Accordingly, PC diagnosis and patient risk stratification are challenging. Both are based mainly on serum prostate-specific antigen (PSA) and histopathologic evaluation of prostate biopsies (most often transrectal ultrasound-guided biopsies (TRUSbx)) or surgical specimens. Hence, there is an urgent need for novel diagnostic and prognostic biomarkers for more personalized PC management.

A new and largely unexplored source for biomarker discovery in PC are circular RNAs (circRNAs) that form a class of primarily noncoding RNAs generated by an alternative splicing event, which covalently links a splice-donor site to an upstream splice-acceptor site [5, 6]. Until recently, circRNAs were viewed as by-products of aberrant RNA splicing events. However, upon the rise of next-generation sequencing, it has now been established that circRNAs can be highly abundant in human cells and can regulate fundamental cellular functions [5, 7, 8]. Thus, circRNAs are implicated in a wide range of physiological and disease processes [1, 9]. Two previous studies have characterized the landscape of circRNA in cancer [10] and in PC in particular [11], demonstrating that circRNA expression is dysregulated in several malignancies, including in PC. Furthermore, circRNAs have higher stability in blood than linear RNAs [5, 12] and are reported enriched in extracellular vesicles (EVs) compared to the level in cells [13, 14], suggesting that EVs could be a compelling source for the discovery of novel minimally invasive circRNA biomarkers. Nonetheless, circRNAs still constitute a poorly characterized output of the human transcriptome and studies of circRNA expression patterns in multiple clinical PC cohorts are limited [10, 11].

Here, we characterize the transcriptomic landscape and biomarker potential of circRNAs in PC by analyzing a cohort of patients with clinically localized or metastatic PC, respectively, and perform independent validation in two additional PC patient cohorts. Finally, we present proof-of-principle of the detection of selected circRNA candidates in EV-enriched plasma samples from PC patients and cancer-free controls.

## Methods

### Patient cohorts

#### Cohort 1 (training)

Cohort 1 included 31 adjacent-normal (AN) and 126 tumor samples from 141 patients with clinically LPC treated by RP (RP cohort 1), as well as 17 primary tumor samples from MPC patients undergoing palliative transurethral resection of the prostate (TURP) (Table 1). All samples were collected at the Department of Urology, Aarhus University Hospital, Denmark (2004–2017) or Department of Urology, Regional Hospital West Jutland, Denmark (2016–2019) (Additional file 1: Supplementary Methods). Inclusion and exclusion criteria for RP cohort 1 are reported according to the REMARK guidelines [15] (Additional file 2: Fig. S1a). circRNA profiling in cohort 1 was performed by total RNA-seq of fresh-frozen (FF) AN and tumor tissue samples (Additional file 1: Supplementary Methods).

**Table 1:** Clinicopathologic characteristics of patient sample sets

Characteristics	Cohort 1 Training (total RNA-seq)		Cohort 2 Validation (NanoString)		RP cohort 3 Validation (NanoString)	Cohort 4 Liquid biopsies (NanoString)	
	RP cohort 1 LPC (*n* = 126)	MPC (*n* = 17)	LPC (*n* = 35)	MPC (*n* = 54)	LPC (*n* = 191)	LPC (*n* = 21)	MPC (*n* = 6)
**Sample type**	RP	TURP	RP (*n* = 22), TRUSbx (*n* = 13)	TRUSbx	RP	Plasma/EVs	Plasma/EVs
**Median age**^**a**^**, years (IQR)**	65.1 (59.2–68.7)	NA	69.4 (62.8–73.0)	71.5 (67.9–74.1)	64.2 (61.5–67.6)	68.4 (64.9–72.8)	73.7 (67.7–74.8)
**Median PSA at diagnosis (IQR)**	10.8 (7.7–17.9)	46.0 (9.7–98.8)	9.2 (6.3–16.5)	30.0 (11.8–46.0)	10.2 (7.1–5.8)	9.1 (6.8–17.2)	NA
**Gleason Grade Group**^**b**^							
1	12 (9.5%)	1 (5.9%)	0	1 (1.9%)	61 (31.9 %)	1 (4.8%)	0
2	68 (54.0%)	1 (5.9%)	13 (37.1%)	1 (1.9%)	105 (55.0 %)	8 (38.1%)	0
3	22 (17.5%)	1 (5.9%)	9 (25.7%)	11 (20.4%)	0	5 (23.8%)	0
4	14 (11.1%)	5 (29.4%)	3 (8.6%)	23 (42.6%)	20 (10.5%)	4 (19.0%)	0
5	9 (7.1%)	8 (47.1%)	10 (28.6%)	17 (31.5%)	5 (2.6%)	2 (9.5%)	3 (50%)
Unknown	1 (0.8%)	1 (5.9%)	0	1 (1.9%)	0	1 (4.8%)	3 (50%)
**T-stage**^**c**^							
T1	0	0	8 (22.9%)	3 (5.6%)	0	4 (19.0%)	1 (16.7%)
T2	75 (59.5%)	2 (11.8%)	12 (34.3%)	15 (27.8)	140 (73.3 %)	13 (61.9%)	3 (50%)
T3	49 (38.9%)	2 (11.8%)	15 (42.9%)	33 (61.1%)	49 (25.7 %)	2 (9.5%)	2 (33.3%)
T4	1 (0.8%)	1 (5.9%)	0	2 (3.7%)	1 (0.3 %)	0	0
Unknown	1 (0.8%)	12 (70.6%)	0	1 (1.9%)	1 (0.5%)	2 (9.5%)	0
**CAPRA-S risk nomogram**							
Low risk	29 (23.0%)	NA	NA	NA	47 (24.6%)	NA	NA
Intermediate risk	60 (47.6%)	NA	NA	NA	100 (52.4%)	NA	NA
High risk	34 (27.0%)	NA	NA	NA	36 (18.8%)	NA	NA
Unknown	3 (2.4%)				8 (4.2%)		
**Surgical margin status**							
Negative	82 (65.1%)	NA	NA	NA	140 (73.3 %)	NA	NA
Positive	41 (32.5%)	NA	NA	NA	51 (26.7 %)	NA	NA
Unknown	3 (2.4%)				0		
**Recurrence status**							
Recurrence-free	75 (59.5%)	NA	NA	NA	108 (56.5 %)	NA	NA
Biochemical recurrence	50 (39.7%)	NA	NA	NA	83 (43.5 %)	NA	NA
Unknown	1 (0.8%)				0		
**Progression to MPC**							
Progression free	NA	NA	NA	NA	180	NA	NA
MPC progression	NA	NA	NA	NA	11	NA	NA
**Median follow-up time, months (IQR)**	65.9 (45.3–102.6)	NA	19.9 (14.2–22.7)	NA	125.3 (98.8–141.7)	NA	NA
**Survival status**							
Alive	110 (87.3%)	NA	NA	NA	155 (81.2%)	NA	NA
Dead	16 (12.7%)	NA	NA	NA	36 (18.8%)	NA	NA
**Non-malignant samples**	AN (*n* = 31, from LPC patients)		AN (*n* = 22, from 20 LPC and 2 MPC patients)			Control (*n* = 27, cancer-free at initial TRUSbx)	
**Median age**^**a**^**(IQR)**	63.4 (58.1–66.5)		68.6 (61.7–71.8)			63.9 (58.5–68.3)	

Data is *n* (%) or median (IQR). ^a^Age at time of sample collection. ^b^For cohort 4, biopsy Gleason Grade Group is stated. ^c^For RP cohorts 1 and 3, pathological T stage is stated and for all other samples, clinical T stage. *IQR* interquartile range, *NA* not available/not applicable

#### Cohort 2 (validation)

Cohort 2 included 22 AN samples, 35 tumor samples from patients with clinically LPC, and 54 primary tumor samples from MPC patients (Table 1, Additional file 1: Supplementary Methods). All patients in cohort 2 were high-risk PC (D’Amico classification [16]) patients referred for primary ^68^Ga-PSMA PET/CT staging at Department of Nuclear Medicine and PET, Aarhus University Hospital (2016-2019).

#### RP cohort 3 (validation)

RP cohort 3 included 191 tumor samples from patients with clinically LPC treated by RP from the Department of Urology, Aarhus University Hospital (1998-2009) (Table 1).

For validation of top circRNA candidates discovered in cohort 1, NanoString-based expression analysis was performed on formalin-fixed paraffin-embedded (FFPE) AN and tumor samples from patients in cohorts 2 and 3 (Additional file 1: Supplementary Methods) [17].

#### Cohort 4 (liquid biopsy analyses)

Cohort 4 included plasma samples from 27 patients histologically verified as cancer-free at initial TRUSbx (controls), 21 patients diagnosed with clinically LPC at initial TRUSbx, and 6 patients with known MPC undergoing palliative TURP (Table 1, Additional file 1: Supplementary Methods). Plasma samples from controls and LPC patients were collected at the Department of Urology, Aarhus University Hospital, Denmark (2017–2020). Plasma samples from MPC patients were collected at Department of Urology, Regional Hospital West Jutland, Denmark (2016–2019). Detection of top circRNA candidates in cohort 4 was performed by NanoString-based expression analysis on EV-enriched plasma samples (Supplementary Methods). Full exclusion criteria for cohorts 2–4 according to the REMARK guidelines [15] can be found in Additional file 2: Fig. S2.

For additional external validation, we downloaded circRNA expression data from the MiOncoCirc database [10] and used the “PRAD” dataset to compare circRNA expression between normal and cancer samples.

All research was carried out in accordance with relevant guidelines and regulations. The studies were approved by The Central Denmark Region Committees on Health Research Ethics [#2000/0299, #1-10-72-361-18, #1-10-72-367-13] and The National Committee on Health Research Ethics [#1603543/66451], and notified to The Danish Data Protection Agency [#2013–41-2041, #1-16-02-330-13, #1-16-02-23-19, #1-16-02-248-14]. For cohorts 1, 3, and 4, written consent was obtained from all participants prior to their donation of blood/tissue samples for a research biobank, while archived tissue was used for cohort 2. In all cases (cohorts 1–4), the requirement for patient consent to the specific analyses in this retrospective study was waived. The research conformed to the principles of the Helsinki Declaration. Biochemical recurrence status for RP cohorts 1 and 3 was updated in September 2020.

### Study design

**Fig. 1:**
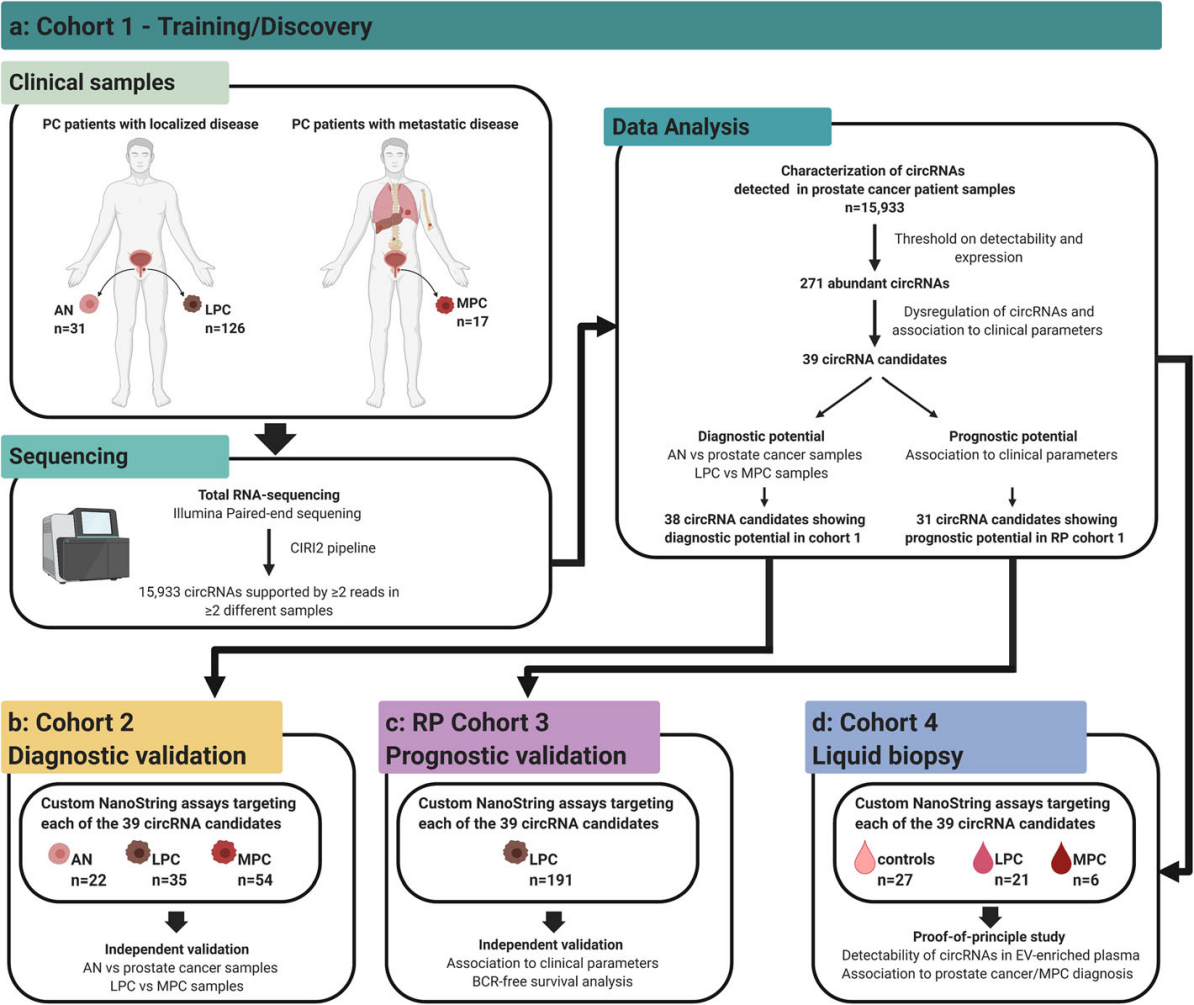
Workflow and patient samples across all four prostate cancer patient cohorts. **a** Cohort 1. **b** Cohort 2. **c** RP cohort 3. **d** Cohort 4. Created with BioRender.com

This study was performed in multiple steps. First, to profile the landscape of circRNAs in non-malignant and PC tissue, we conducted transcriptome-wide expression profiling in cohort 1 (training, Fig. 1a), consisting of 31 AN and 126 tumor samples from patients with LPC (RP cohort 1) as well as 17 primary tumor samples from MPC patients. Next, using cohort 1, we evaluated the diagnostic and prognostic biomarker potential for circRNAs and selected 39 top candidate circRNAs for independent validation in two additional patient cohorts, including 22 AN, 35 LPC, and 54 MPC samples (cohort 2) and 191 LPC samples (RP cohort 3), respectively (Fig. 1b,c). Finally, as proof-of-principle, we tested the biomarker potential in liquid biopsies for the 39 circRNA candidates using EV-enriched plasma samples from 27 cancer-free controls, 21 LPC, and 6 MPC patients (cohort 4; Fig. 1d).

### Total RNA sequencing and circRNA quantification

Transcriptome-wide RNA-seq libraries were prepared from total RNA after depletion of rRNA using the Ribo-Zero^TM^ Magnetic Gold Kit (Epicentre, an Illumina company) or KAPA RiboErase Kit (Roche). Library preparation was performed using the ScriptSeq RNA-Seq Library Kit (Epicentre) or KAPA RNA HyperPrep Kit (Roche). All libraries were sequenced paired-end on either an Illumina HiSeq 2000 (2 × 75 base pairs (bp)), NextSeq 500 (2 × 75 bp), or NovaSeq 600 (2 × 100 bp) with a coverage target of 25 million reads/sample.

Sequencing data from samples in cohort 1 were analyzed using CIRI2 [18], described as the best stand-alone bioinformatics algorithm for circRNA quantification [19]. circRNAs were annotated according to hg19 and default thresholds for mapping quality of each segment of junction reads (10), and maximum spanning distance of a circRNA (200,000) was applied. Only circRNAs supported by more than two back-splice junction (BSJ)-spanning reads in at least two different samples were included in the final data analysis. The length of individual circRNAs was estimated by calculating the difference between exon start and exon end in a BSJ, which may fail to capture changes in length due to exon-intron circRNAs [20] or circRNAs effected by alternative splicing [21].

Identified BSJ reads were normalized to the total number of reads in each sample and log2 transformed using the cpm function in the R package edgeR (v3.30.3) [22]. A batch correction was performed due to the use of two different library preparation kits, using the removeBatchEffect function in the Limma package (v3.44.3) [23]. Circular-to-linear ratios (circ/lin) were calculated by CIRI2 [18] by dividing the number of reads spanning a particular BSJ by the corresponding number of reads spanning the same splice site, but consistent with linear RNA instead of being back-spliced. To avoid division by zero, a pseudocount of one was added to both the number of BSJ reads and the number of linear reads.

### NanoString nCounter Codeset design and circRNA expression analysis

A custom CodeSet of capture and reporter probes was designed to target regions of 100 nucleotides overlaying the BSJ of 39 selected top candidate circRNAs identified in cohort 1 (Additional file 3: Table S1). In addition, five reference transcripts were included, identified as stably expressed in the total RNA-seq data by the NormFinder algorithm [24] (ACTB, HPRT1, RPS24, circARHGAP12, and circRBM23).

For FFPE and plasma samples, 300 ng and 5 μL total RNA, respectively, was subjected to nCounter™ SPRINT (NanoString Technologies) analysis according to the manufacturer’s instructions. Hybridization time was set to 23 h. Background subtraction and subsequent normalization was performed using the nSOLVER 3.0 software (NanoString Technologies). A background threshold of 10 was selected, which all raw counts at or below were set to. Normalization was performed using the geometric mean of the five reference transcripts (ACTB, HPRT1, RPS24, circARHGAP12, and circRBM23).

### Statistical analyses

All statistical analyses were conducted in R (version 3.6.1) using R Studio version 1.3.959. The 100 circRNAs with the greatest variance across cohort 1 were selected among the most abundant circRNAs (*n* = 271) and subject to non-negative matrix factorization (NMF) consensus clustering using the package NMF (v0.23.0) [25]. The optimal number of clusters was determined using the rank-estimation function in the NMF package, testing ranks 2–6. Consensus clustering with 5000 iterations was performed.

Comparison between different sample types and/or patient subgroups was performed using the non-parametric Wilcoxon rank-sum test or Kendall’s rank correlation. The diagnostic potential of circRNAs was evaluated by receiver operating characteristics (ROC) curve analysis between AN and PC samples.

The prognostic potential of circRNAs was evaluated by Kaplan-Meier, uni- and multivariate Cox regression analyses using the survival package (v3.1.12) [26] with postoperative BCR (PSA ≥ 0.2 ng/mL) or progression to MPC (defined by medical journal entry) as clinical endpoints. All circRNAs that did not fulfill the proportional hazard assumption (tested by the cox.zph function in the survival package) were removed. Patients not having experienced BCR or progression to MPC after RP were censored at their last normal PSA test. For survival analyses, patients in RP cohort 1 (training) were dichotomized according to circRNA expression levels based on the optimal cut-off identified by ROC analysis of BCR status, using Youden’s J statistic in the pROC package (v1.16.2) [27]. The cut-off fraction for circITGA7, circKDM1A, and the 5-circRNA signature identified in RP cohort 1 (0.70, 0.66, and 0.68, respectively) was subsequently used and tested in RP cohort 3. In multivariate Cox regression analysis, comparisons to the CAPRA-S risk nomogram were performed [28]. *P* values were adjusted for multiple testing using the Benjamini-Hochberg (BH) approach and considered significant if below 0.05.

## Results

### Profiling of circRNAs in prostate cancer

**Fig. 2:**
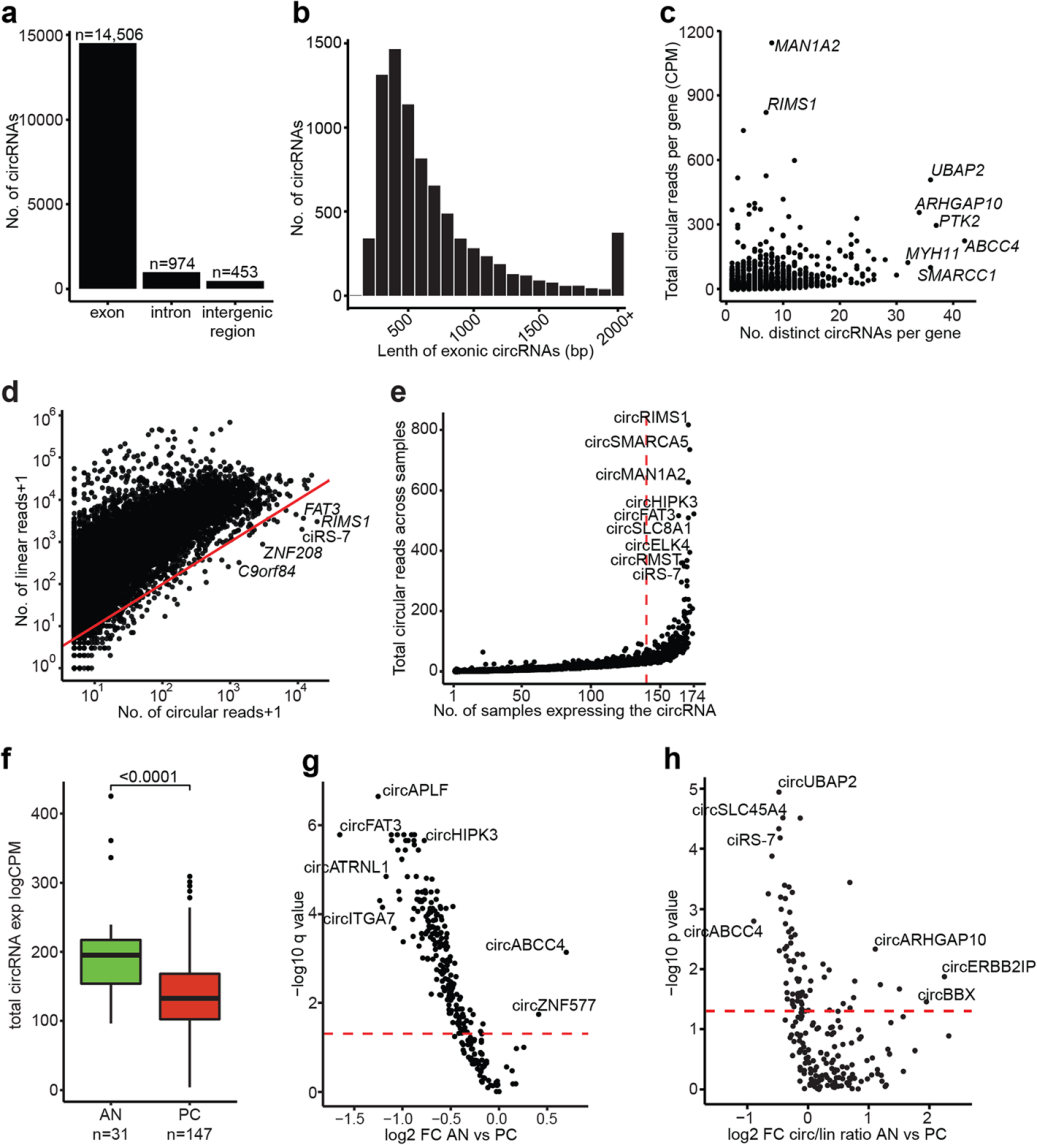
Profiling of circRNAs in prostate cancer patients. **a** Genomic origin of circRNAs. **b** Estimated exonic length of circRNAs. Bin size = 100 bp. **c** Number of total circular reads (CPM) per gene versus number of distinct circRNAs per gene. **d** For each circRNA, the number of circular and corresponding linear reads on a logarithmic scale. Above the red line: Linear > circRNA, below the red line: circRNA > linear. **e** Total number of circular reads across all patient samples *vs*. the number of samples expressing each distinct circRNA. The red dotted line marks circRNAs detected in more than 80% of all samples. **f** Boxplot of total expression (CPM) of abundant circRNA across cancer (LPC and MPC) and AN samples in cohort 1. *P* value represents Wilcoxon rank-sum test. **g–h** Volcano plot of (**g**) abundant circRNAs or (**h**) circ/lin ratio for abundant circRNAs showing log2 fold change between cancer and AN samples in cohort 1 according to the levels of significance. Horizontal dashed line corresponds to q (**g**) or p (**h**) = 0.05. X-axis and Y-axis are plotted on a logarithmic scale (log2 and log10, respectively). CPM = counts per million. FC = fold change

For unbiased profiling of circRNA expression in AN and PC tissue samples, we analyzed 31 AN and 126 tumor samples from patients with LPC as well as 17 primary tumor samples from MPC patients by total RNA sequencing (cohort 1, training). Using the CIRI2 pipeline on the complete RNA-seq data set from cohort 1 (*n* = 174 samples), we identified 27,458 unique circRNAs supported by at least two BSJ spanning reads. Of these, 11,525 circRNAs were only supported by a single sample and hence excluded, leaving 15,933 circRNAs eligible for further analysis (Fig. 1a). The majority (91.0%) of the identified 15,933 circRNAs originated from protein-coding exons (Fig. 2a) and had a median estimated length of 484 bp (Fig. 2b). This is in agreement with previous reports for human exonic circRNAs [1, 29]. The remaining circRNAs originated from introns (6.1%) and intergenic regions (2.9%) (Fig. 2a), suggesting that a minority of cirRNAs expressed in prostatic tissue derive from ncRNA.

There was profound variation in the number of uniquely expressed circRNAs per gene. The majority of circRNA host genes (4849/5061; 96%) gave rise to one to ten unique circRNAs, while a few genes (7/5061; 0.1%) gave rise to over 30 unique circRNAs (*ABCC4*, *ARHGAP10*, *BIRC6*, *MYH11*, *PTK2*, *SMARCC1*, and *UBAP2*; Fig. 2c). A subset of circRNAs (962/15,933; 5.9%) were more highly expressed than their linear counterpart, e.g., circRIMS1, ciRS-7, and circFAT3 (Fig. 2d), indicating differential regulation of the expression of specific circular transcript as compared to their linear counterparts.

We evaluated the abundance of the 15,933 detected circRNAs across all 174 samples from cohort 1 and identified a subset of 268 circRNAs (1.7%) expressed in > 80% of all samples analyzed (Fig. 2e). As biomarker candidates must be robustly detectable to be of clinical use, we limited all further analyses to a set of 271 abundant circRNAs, which were either expressed in > 80% of all samples and/or belonging to the top 1% most highly expressed circRNAs in cohort 1 (*n* = 159, based on ranked mean expression) (Fig. 1a).

### Dysregulated circRNAs in prostate cancer

Using unsupervised consensus clustering of circRNA-based expression values, we identified two stable clusters (Additional file 2: Fig. S3a) that almost completely separated AN and MPC samples, but not LPC from AN or MPC, respectively (Additional file 2: Fig. S3b). The two clusters showed significant differences in their composition, with cluster 1 being enriched for MPC samples and advanced stage LPC samples (*P* = 0.003 and *P* = 0.03, respectively, chi-square test), while cluster 2 was enriched for AN samples (*P* < 0.0001, chi-square test) (Additional file 2: Fig. S3b-d). Consistent with this, cluster 1 was moderately enriched for higher-grade LPC samples, although this was not statistically significant (*P* = 0.25, chi-square test, Additional file 2: Fig. S3e). Cluster 1 was also significantly associated with lower overall expression of circRNAs (*P* < 0.0001, Wilcoxon rank-sum test, Additional file 2: Fig. S3f), together suggesting a possible association between low circRNA expression and clinical parameters associated with more aggressive PC.

Similarly, differential expression analysis of all 271 abundant circRNAs showed a global downregulation of circRNA levels in PC (LPC + MPC) as compared to benign (AN) prostate tissue samples (*P* < 0.0001, Fig. 2f). Overall, in cohort 1, we identified 204 circRNAs that were significantly downregulated and two circRNAs that were significantly upregulated in PC *vs.* AN samples (*P* < 0.05, BH-adjusted Wilcoxon rank-sum test, Fig. 2g, Additional file 4: Table S2a). In terms of fold change, the most significantly downregulated circRNAs in cohort 1 were circFAT3 (3.13-fold, *P* < 0.0001), while the most upregulated circRNA was circABCC4 (1.62-fold, *P* = 0.001) (Fig. 2g). Out of the 206 dysregulated circRNAs, a total of 71 (34%) also showed a significant change in circ/lin ratio between PC *vs.* AN samples (53 in the same direction, *P* < 0.05, Wilcoxon rank-sum test, Additional file 4: Table S2a), including top candidates circFAT3 and circABCC4.

Similar results were obtained for differential expression analyses of circRNA levels and circ/lin ratios in subgroup comparisons of AN *vs*. LPC and AN *vs*. MPC, respectively (Additional file 4: Table S2b). Furthermore, we identified 52 circRNAs as significantly deregulated between LPC and MPC samples (1 upregulated and 51 downregulated in MPC, respectively, *P* < 0.05, BH-adjusted Wilcoxon rank-sum test, Additional file 4: Table S2a). Of these, 33 circRNAs displayed a significant gradual downregulation from AN to LPC to MPC samples (*P* < 0.05, BH-adjusted Wilcoxon rank-sum test, Additional file 4: Table S2). circFAT3 presented as the most significantly downregulated circRNA, in terms of fold change, in both LPC *vs*. AN (2.52-fold, *P* < 0.0001) and MPC *vs*. LPC (6.46-fold, *P* < 0.0001) (Additional file 4: Table S2).

### Identification and selection of circRNA candidates for independent validation

To identify candidate circRNAs with diagnostic/prognostic biomarker potential in cohort 1 (training), we used the results of differential expression analyses of AN *vs*. PC samples (see above and Additional file 4: Table S2a) combined with additional analyses of clinically relevant subgroups of LPC patients (RP cohort 1). The latter included association analyses between circRNA expression and key clinicopathological parameters known to be linked with PC aggressiveness: pT stage, Gleason Grade Group (GG), and BCR status (Additional file 5: Table S3 and text below).

The best performing circRNAs from each analysis in cohort 1 (ranked by P value) were selected, while also favoring circRNAs significant in more than one analysis and taking expression level into consideration. Based on this, we selected 39 top diagnostic and/or prognostic circRNA candidates (Additional file 3: Table S1) for validation in two independent PC patient sets (cohort 2 and RP cohort 3, Fig. 1b,c). Independent validation was performed using NanoString assays targeting each of these 39 circRNAs. Cohort 2 comprised of 22 AN, 35 LPC, and 54 MPC samples (RP/TRUSbx specimens) and was used to validate top diagnostic candidate circRNAs. Due to incomplete clinical follow-up, cohort 2 was not eligible for prognostic analyses. RP cohort 3 included 191 LPC patients with 125 months median follow-up (Table 1) and was used for validation of prognostic circRNA candidates associated with clinicopathological parameters and/or BCR.

### Validation of dysregulated circRNA candidates in prostate cancer

In cohort 1, a total of 38 selected top candidate circRNAs were significantly deregulated between PC and AN samples (2 upregulated and 36 downregulated in PC; *P* < 0.05, BH-adjusted Wilcoxon rank-sum test, Additional file 6: Table S4a). Of these, we successfully validated 37 (97%) in cohort 2 (2 upregulated and 35 downregulated, *P* < 0.05, BH-adjusted Wilcoxon rank-sum test, Additional file 6: Table S4a). Similar results were obtained by subgroup analyses (AN *vs.* LPC or AN *vs.* MPC, see Additional file 6: Table S4b-c). Next, using the MiOncoCirc database [10] for further external validation, we could find 33 of the 38 circRNAs and validated 28 (85%) of them as significantly dysregulated between PC (*n* = 323) and AN (*n* = 17) samples (all downregulated in PC; BH-adjusted Wilcoxon rank-sum test, Additional file 6: Table S4a).

**Fig. 3:**
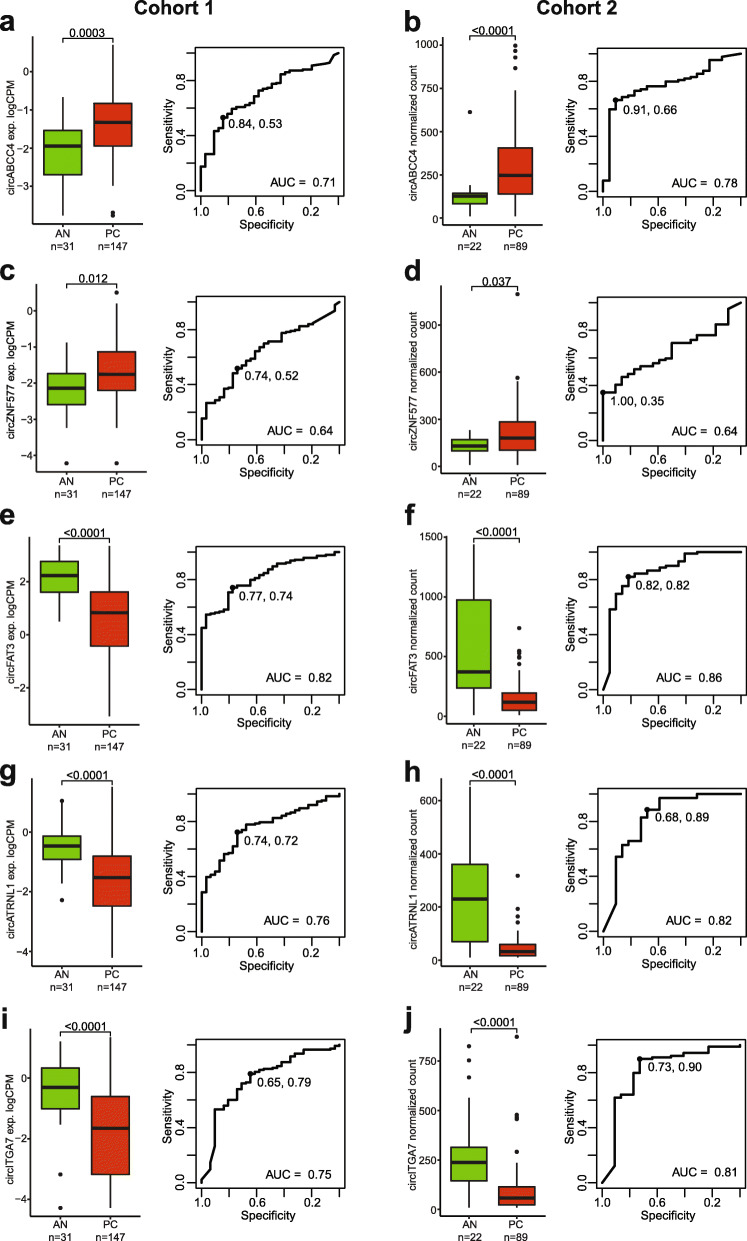
Individual circRNAs with diagnostic potential in prostate cancer. Boxplot (left) of individual circRNA expression across AN and cancer (LPC and MPC) patient tissue samples (**a, c, e, g, i**: cohort 1; **b, d, f, h, j**: cohort 2). *P* values represent Wilcoxon rank-sum test. Boxes represent the 25th and 75th percentiles and median. Outlier cases, defined as more than 1.5 times the IQR from the median are marked as individual dots outside the whiskers. ROC curve analysis (right) for distinguishing PC from AN tissue specimens. Specificity and sensitivity at optimal cut-off are shown

The most significantly upregulated circRNAs in PC tissue samples were circABCC4 and circZNF577, while the most significantly downregulated were circFAT3, circITGA7, and circATRNL1 (ranked by fold change in cohort 1, Fig. 3, Table 2). ROC curve analyses showed that four of these five circRNAs (circABCC4, circFAT3, circATRNL1, and circITGA7) had AUCs above 0.7, ranging from 0.71 to 0.82 in cohort 1 and 0.78 to 0.86 in cohort 2 (Fig. 3), suggesting promising diagnostic potential for PC. A logistic regression model using a combination of the five circRNAs did not yield a significantly (bootstrap *p* > 0.05) higher AUC than the best single circRNA (data not shown).

**Table 2:** Successfully validated dysregulated circRNAs in prostate cancer

	Cohort 1			Cohort 2		
**Upregulated in PC** ***vs.*** **AN**	**Adj.** ***P*** **value**	**FC**	**AUC**	**Adj.** ***P*** **value**	**FC**	**AUC**
circABCC4	0.0004	1.62	0.71	< 0.0001	2.31	0.78
circZNF577	0.01	1.33	0.64	0.04	1.56	0.64
**Downregulated in PC*vs. *AN**	**Adj.*P *value**	**FC**	**AUC**	**Adj.*P *value**	**FC**	**AUC**
circFAT3	< 0.0001	− 3.14	0.82	< 0.0001	− 3.79	0.86
circITGA7	< 0.0001	− 2.31	0.75	< 0.0001	− 2.96	0.81
circATRNL1	< 0.0001	− 2.25	0.78	< 0.0001	− 5.54	0.85
circSLC45A4	< 0.0001	− 2.17	0.82	< 0.0001	− 1.96	0.85
circRNASEH2B	< 0.0001	− 2.08	0.81	< 0.0001	− 2.00	0.81
circSEMA3C	< 0.0001	− 2.07	0.80	< 0.0001	− 2.25	0.82
circSLC8A1	< 0.0001	− 2.04	0.76	< 0.0001	− 2.12	0.87
circARHGAP10	< 0.0001	− 2.01	0.79	< 0.0001	− 1.92	0.83
**Downregulated in MPC*vs. *LPC**	***P *value**	**FC**	**AUC**	***P *value**	**FC**	**AUC**
circMKLN1	0.0004	− 2.03	0.77	0.028	− 1.36	0.64
circN4BP2L2	0.03	− 1.77	0.67	< 0.0001	− 1.53	0.76
circZNF532	0.01	− 1.76	0.69	0.0002	− 1.78	0.74
circCDYL2	0.04	− 1.60	0.66	< 0.0001	− 2.19	0.76
circARHGAP10	0.001	− 2.13	0.76	0.001	− 1.74	0.71

Results for the top dysregulated candidates in PC *vs.* AN and in MPC *vs.* LPC, respectively. For upregulated circRNAs in PC, all candidates significant in cohort 1 are shown. For downregulated circRNAs in PC, candidates with fold change > − 2 in cohort 1 are shown. For dysregulated circRNAs in LPC *vs.* MPC, candidates significant (uncorrected *P* < 0.05) in both cohorts 1 and 2 are shown (all downregulated)

Out of the 38 circRNAs dysregulated in PC *vs.* AN samples in cohort 1, a total of 21 circRNAs were also significantly deregulated between MPC and LPC samples in the same cohort (*P* < 0.05, all downregulated in MPC, Wilcoxon rank-sum test, Additional file 6: Table S4a). When comparing MPC *vs*. LPC samples in cohort 2, we could validate five (24%) of these circRNAs (circMKLN1, circN4BP2L2, circZNF532, circCDYL2, and circARHGAP10, *P* < 0.05, Wilcoxon rank-sum test, Table 2, Additional file 2: Fig. S4).

### Association of abundant circRNAs to clinicopathological parameters of prostate cancer aggressiveness

In RP cohort 1 (training, *n* = 126 LPC samples), we found 129 abundant circRNAs that were significantly associated with either advanced pT stage (Additional file 5: Table S3a), high GG (Additional file 5: Table S3b), or BCR status (Additional file 5: Table S3c), suggesting possible prognostic biomarker potential. Of these, the 31 most promising candidates were selected for further testing in RP cohort 3, as described above (Fig. 1a,c, Additional file 7: Table S5).

Among the 31 circRNA candidates with prognostic potential, a total of 24 were significantly deregulated between high/low pT stage in RP cohort 1 (all downregulated in pT2 *vs.* pT3-4, *P* < 0.05, Wilcoxon rank-sum test, Additional file 7: Table S5a). We successfully validated five (21%) of these (circZNF532, circCDYL2, circLPAR3, circELK4, and circMAN1A2) in RP cohort 3 (*P* < 0.05, Additional file 2: Fig. S5, Table 3). Furthermore, 17 of the 31 circRNA candidates were significantly correlated to GG in RP cohort 1 (all negatively correlated, *P* < 0.05, Kendall’s rank correlation, Additional file 7: Table S5b). We successfully validated three (18%) of these circRNAs in RP cohort 3 (circSLC45A4, circFAT3, and circSEMA3C; *P* < 0.05, Table 3, Additional file 2: Fig. S5).

**Table 3:** circRNA candidates validated as significantly associated with prostate cancer aggressiveness in both cohorts

	pT2 ***vs***. pT3-4				Correlation to GG				BCR ***vs.*** recurrence-free			
	RP Cohort 1		RP Cohort 3		RP Cohort 1		RP Cohort 3		RP Cohort 1		RP Cohort 3	
circRNA	***P*** value	FC	***P*** value	FC	***P*** value	Tau	***P*** value	Tau	***P*** value	FC	***P*** value	FC
circMKLN1	**0.01**	− 1.34	0.58	− 1.11	0.21	− 0.08	**0.03**	− 0.13	**0.001**	− 1.41	**0.04**	− 1.30
circZNF532	**0.002**	− 1.55	**0.001**	− 1.35	0.08	− 0.12	0.14	− 0.08	**0.01**	− 1.43	0.23	− 1.12
circMAN1A2	**0.003**	− 1.36	**0.03**	− 1.12	**0.03**	− 0.15	0.36	− 0.05	0.15	− 1.22	0.85	1.00
circSEMA3C	**0.001**	− 1.55	0.77	1.00	**0.01**	− 0.17	**0.03**	− 0.13	0.28	− 1.25	0.85	1.04
circLPAR3	**0.01**	− 1.46	**0.01**	− 1.40	**0.03**	− 0.15	0.31	− 0.06	0.37	− 1.24	0.11	− 1.12
circCDYL2	**0.04**	− 1.17	**0.01**	− 1.33	**0.03**	− 0.14	0.21	− 0.07	0.09	− 1.26	0.19	− 1.15
circELK4	**0.02**	− 1.31	**0.01**	− 1.33	0.06	− 0.13	**0.03**	− 0.12	0.11	− 1.19	0.22	− 1.18
circFAT3	**0.01**	− 1.60	0.06	− 1.47	**0.01**	− 0.17	**0.01**	− 0.15	0.22	− 1.20	0.05	− 1.25
circSLC45A4	**0.01**	− 1.49	0.61	− 1.01	**0.003**	− 0.20	**0.02**	− 0.13	0.42	− 1.09	0.10	− 1.17

In RP cohort 1, seven out of 31 candidate circRNAs were significantly deregulated between patients with or without BCR (all downregulated in patients with BCR, *P* < 0.05, Wilcoxon rank-sum test, Additional file 7: Table S5c). We successfully validated one of these circRNAs (circMKLN1) in RP cohort 3 (*P* = 0.04, Wilcoxon rank-sum test, Table 3, Additional file 2: Fig. S5).

In summary, we validated nine of the 31 prognostic circRNA candidates associated with key clinicopathological parameters (pT stage, GG, and BCR status) of PC aggressiveness identified in RP cohort 1 in the independent RP cohort 3 (Table 3).

### Prognostic performance of individual circRNA candidates

Using circRNA expression as a continuous variable in univariate Cox regression analysis of time to BCR, 11 of the 39 circRNA candidates were significant in RP cohort 1 (uncorrected *P* < 0.05, Additional file 8: Table S6). Of these 11, three circRNAs (circITGA7, circMKLN1, and circTULP4) were independently validated in RP cohort 3 (univariate cox regression; *P* < 0.05, HR 0.64 to 0.79, Additional file 8: Table S6). When analyzed as continuous variables, two of the three circRNAs (circMKLN1 and circTULP4) remained significant predictors of BCR-free survival after adjustment for the clinical nomogram CAPRA-S, but only in RP cohort 3 (*P* < 0.05, Additional file 8: Table S6).

However, when applied clinically, biomarkers are often preferred as dichotomous variables for easy interpretation of test results. Using RP cohort 1 for training, we divided patients into high and low circRNA expression groups based on ROC analysis of BCR status for each of the 39 circRNA candidates. We identified 24 circRNAs significant in both Kaplan-Meier and univariate analysis in RP cohort 1 (uncorrected *P* < 0.05, Additional file 8: Table S6), of which we independently validated circKDM1A and circITGA7 in RP cohort 3 using the cut-off fraction trained in RP cohort 1.

**Fig. 4:**
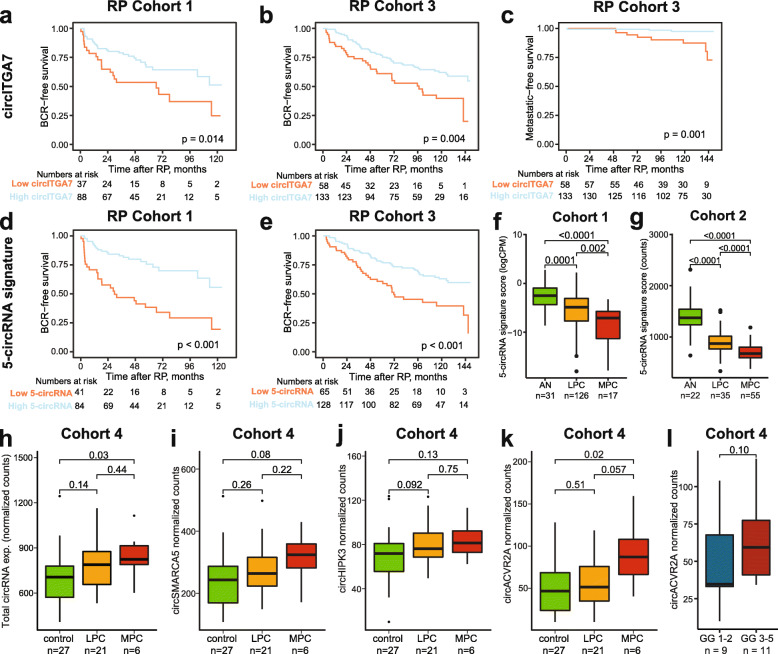
circRNA candidates hold strong prognostic potential and are detectable in EV-enriched plasma samples. **a–e** Kaplan-Meier analysis of biochemical recurrence (BCR)-free survival (**a,b,d,e**) or progression to MPC (**c**) in RP cohort 1 (**a,d**) and RP cohort 3 (**b,c,e**). Patients in RP cohorts 1 and 3 were dichotomized based on cut-off trained in RP cohort 1 from circITGA7 expression (**a–c**) or the 5-circRNA signature (**d, e**). For each Kaplan-Meier plot, *p* values for two-sided log-rank tests and the number of patients at risk are given. **f, g** Boxplot of 5-circRNA signature score in cohort 1 (**f**) and cohort 2 (**g**). **h–k** Boxplot of overall (**h**) or individual **(i–k)** circRNA levels in EV-enriched plasma samples across sample types (cohort 4, *n* = 54). **l** circACVR2A expression across GG in diagnostic biopsies from LPC patients. Boxes represent the 25th and 75th percentiles and median. Outlier cases, defined as more than 1.5 times the IQR from the median are marked as individual dots outside the whiskers

For both of these circRNAs, low expression was significantly associated with BCR (Fig. 4a,b, Additional file 2: Fig. S6, Additional file 8: Table S6). Thus, in univariate analyses, circITGA7 was a significant predictor of BCR in two independent RP cohorts, both as a continuous and a dichotomous variable.

After adjustment for the CAPRA-S nomograms in multivariate analysis, only circITGA7 remained significant and only in RP cohort 3 (*P* = 0.03, HR 1.64, 95% CL 1.04–2.58, Additional file 8: Table S6). Notably, low circITGA7 (but not circKDM1A) expression was also significantly associated with progression to MPC in both Kaplan-Meier and univariate cox regression analysis in RP cohort 3 (*P* = 0.005, HR 6.8, 95% CI 1.8–25.6, univariate Cox regression, Fig. 4c), where long patient follow-up allowed for investigation of this additional endpoint (> 10 years). Due to low event numbers (*n* = 11), multivariate analysis of metastasis-free survival was not feasible.

### Development of a prognostic 5-circRNA signature to independently predict postoperative BCR

Next, we tested if a multi-marker signature could improve prognostic accuracy. Therefore, the five circRNAs (as dichotomous variables) with the strongest individual association to BCR in univariate Cox regression analysis in RP cohort 1 (based on *P* value and HR *P* < 0.0002 and HR > 2.6, Additional file 8: Table S6), were combined in a 5-circRNA prognostic signature (Sum_logCPM_(circKMD1A, circTULP4, circZNF532, circSUMF1, circMKLN1)). In RP cohort 1, a low 5-circRNA signature score was significantly associated with BCR in both Kaplan-Meier and univariate Cox regression analysis (dichotomous, HR = 3.3, *P* < 0.0001, Fig. 4d, Table 4). The 5-circRNA signature remained significant in multivariate Cox regression analysis after adjusting for the CAPRA-S nomogram and increased the predictive accuracy (C-index) from 0.71 to 0.75 (Table 4).

**Table 4:**
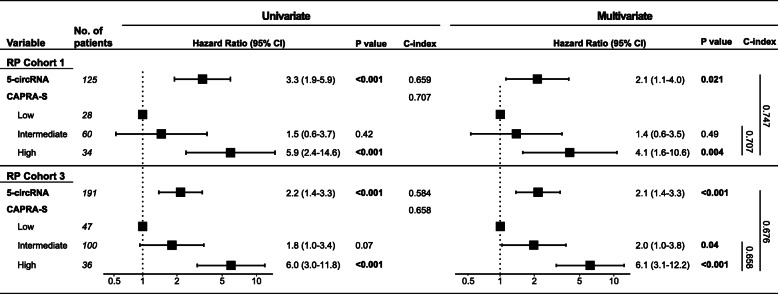
Uni- and multivariate Cox regression analysis of BCR using 5-circRNA prognostic signature

Uni- and multivariate Cox regression analyses of BCR in RP cohort 1 (*n* = 125, 42 events) and RP cohort 3 (*n* = 191, 83 events). *CI* confidence interval, *C-index* Harrell’s concordance index

Using the same cut-off fraction as trained in RP cohort 1, the 5-circRNA signature was successfully validated in RP cohort 3 as a significant predictor of BCR in Kaplan-Meier, uni- and multivariate analysis (*P* < 0.05, Fig. 4e, Table 4). In multivariate analysis, the 5-circRNA signature increased the C-index from 0.66 to 0.68 when combined with the CAPRA-S nomogram. The 5-circRNA signature was not significantly associated with progression to MPC in univariate analysis in RP cohort 3. However, the 5-circRNA signature score was significantly lower in MPC *vs.* LPC as well as in LPC *vs.* AN samples in both cohorts 1 and 2 (*P* < 0.05, Wilcoxon rank-sum test, Fig. 4f,g).

Taken together, these results suggest that combining the five circRNAs with the strongest individual prognostic potential into a simple dichotomous signature can improve robustness compared to a single circRNA. The 5-circRNA prognostic signature may refine the prediction of BCR after surgery beyond routine clinicopathological variables (Table 4).

### Detectability of circRNA candidates in EV-enriched plasma samples from prostate cancer patients and controls

As proof-of-principle, we tested whether the 39 top circRNA biomarker candidates were detectable in liquid biopsies. We analyzed EV-enriched plasma samples from 27 cancer-free controls, 21 LPC patients, and 6 MPC patients (cohort 4, *n* = 54; Table 1, Fig. 1d) using the custom NanoString assays. Of the 39 circRNA candidates, 23 (59%) were detectable in EV-enriched plasma samples. The 16 circRNAs not detected in EV-enriched plasma samples tended to be more lowly expressed in tumor and AN prostate tissue samples (cohorts 1 and 2, Additional file 2: Fig. S7a-d).

For the 23 circRNAs detected in EV-enriched plasma, we identified a significant higher overall level of circRNAs from control to MPC samples (*P* = 0.03, Fig. 4h). Five circRNAs (circSMARCA5, circHIPK3, circACVR2A, circN4BP2L2, and circMAN1A2) were particularly abundant in EV-enriched plasma (detected in > 89% of all samples; Additional file 9: Table S7). Three of these (circSMARCA5, circHIPK3, and circMAN1A2) were also among the top five most highly expressed circRNA candidates in patient tumor and AN tissue samples (cohort 1), whereas circACVR2A was among the most lowly expressed (Additional file 3: Table S1), combined, indicating both substantial correlations in circRNA levels between tissue and EV-enriched plasma, respectively, as well as some differences.

The level of circSMARCA5, circHIPK3, and circACVR2A in EV-enriched plasma samples gradually increased from control to LPC to MPC patients (Fig. 4i–k). Contrary, in patient tissue samples, expression levels for these three circRNAs gradually decreased from AN to LPC to MPC samples in cohorts 1 and 2 (Additional file 2: Fig. S7e-j).

circACVR2A levels were significantly elevated in EV-enriched plasma from MPC patients relative to controls (*P* = 0.02, Fig. 4k). Interestingly, circACVR2A levels were also moderately elevated in EV-enriched plasma samples from LPC patients with high-grade tumors (GG 3–5) in initial TRUSbx compared to LPC patients with low-grade tumors (GG 1–2) in cohort 4 (*P* = 0.10, Fig. 4l), highlighting the possible potential of circRNAs as minimally invasive biomarkers.

## Discussion

This work represents one of the largest and most comprehensive circRNA profiling studies in PC to date. We have described the transcriptomic landscape of circRNAs in AN and tumor tissue samples from patients with localized and metastatic PC, and defined a novel set of 271 abundantly expressed circRNA in benign/malignant prostatic tissue. In addition, we profiled the biomarker potential of this set of abundant circRNAs and selected a set of 39 top candidates for validation using NanoString-based expression analysis in two independent PC patient cohorts (cohort 2, *n* = 111; RP cohort 3, *n* = 191). Lastly, we demonstrated the detectability of a subset of these circRNA candidates in EV-enriched plasma samples from PC patients and cancer-free controls, highlighting the promising future potential of circRNAs also as minimally invasive biomarkers.

We identified 15,933 unique circRNAs across a total of 174 AN and PC tumor samples (cohort 1). Consistent with previous findings in other tissue types, most circRNAs in PC were spliced from protein-coding genes, with the majority (96%) giving rise to one to ten circRNA isoforms [1, 6].

Investigating differentially expressed circRNAs in PC, we found a significant global downregulation of abundant circRNAs in PC compared to AN samples in cohort 1 (*P* < 0.0001, Fig. 2f). This is in agreement with previous findings in other cancer types, where circRNAs have been described as generally downregulated in tumor compared to AN/control tissue samples [10, 30–32].

In addition, we identified and validated circABCC4, circFAT3, circATRNL1, and circITGA7 as possible novel diagnostic candidate circRNA biomarkers specific for PC (AUCs ranging from 0.71 [circABCC4, cohort 1] to 0.86 [circFAT3, cohort 2]), and especially, circFAT3 showed promising diagnostic potential (Table 2, Fig. 3e–f). Here, we identified a significant upregulation of circABCC4 in PC, confirming and expanding on results from recent studies using 47 PC and 47 paired AN samples [33] as well as 25 PC and 25 paired AN samples [10]. Consistent with this, it has been reported that circABCC4 promotes PC cell line proliferation, cell-cycle progression, migration, and invasion, possibly by regulating FOXP4 expression through sponging of miR-1182 [33]. None of the other three circRNAs (circFAT3, circATRNL1, and circITGA7) have been functionally linked to PC or described in expression studies in PC patient samples. However, both circITGA7 and circATRNL1 have been described as downregulated in other cancers, including colorectal cancer (CRC) (circITGA7, [34, 35]) and ovarian cancer (circATRNL1, [36]), similar to our findings in PC. Moreover, circITGA7 and circATRNL1 have been reported to be involved in cell proliferation, invasion, and migration in CRC and ovarian cancer [34, 36], supporting a potential functional role of these circRNA candidates in cancer development/progression. circFAT3 has not previously been described in relation to cancer. However, the FAT family genes, including *FAT3*, has been described to be involved in tumor suppression [37]. Further studies should investigate the function of these circRNAs in relation to PC biology.

Investigating prognostic biomarker potential, we found low circITGA7 expression to be significantly associated with BCR in Kaplan-Meier and univariate Cox regression analysis in both RP cohorts 1 and 3 (Fig. 4a,b, Additional file 8: Table S6), as well as with shorter time to MPC progression in RP cohort 3 (Fig. 4c, Additional file 8: Table S6). These findings support possible tumor-suppressive properties of circITGA7 in PC and are further supported by results in CRC, where low circITGA7 expression has been reported to be associated with progression in CRC patients [34]. Nonetheless, we were not able to validate any single circRNA candidates in multivariate analysis adjusting for established clinicopathologic variables, suggesting that the molecular heterogeneity of PC requires multiple circRNAs to hold sufficient robustness.

Accordingly, we developed and validated a novel 5-circRNA prognostic signature for PC (circKMD1A, circTULP4, circZNF532, circSUMF1, and circMKLN1). To the best of our knowledge, this is the first report of a prognostic circRNA signature for PC with significant independent prognostic value in multiple distinct PC patient cohorts. A low 5-circRNA signature score significantly predicted time to BCR in multivariate analysis including established clinicopathologic variables in both RP cohorts 1 and 3 (Table 4), indicating that this novel 5-circRNA signature holds independent prognostic value for PC. Accordingly, these results suggest that the detection of circRNAs in tissue specimens from LPC patients could potentially be utilized in the future for guiding decisions regarding adjuvant treatment after RP as a predictor of risk of BCR. The 5-circRNA signature could potentially aid the identification of patients who would benefit from intensified treatment, e.g., radiotherapy and/or androgen deprivation therapy (ADT), following RP to reduce the risk of BCR. Further validation is warranted.

None of the circRNAs in our novel 5-circRNA prognostic signature have been investigated in relation to cancer before, highlighting an unused potential of the circRNA output from the human transcriptome. The host genes of the five circRNAs are functionally diverse: KDM1A is a histone demethylase and has been demonstrated to mediate transcriptional activation of the androgen receptor [38], which is associated with PC initiation and progression to castration-resistant prostate cancer [39]. KDM1A inhibition has also been reported to suppress PC tumor growth in male mice [40]. The oxidase SULF1 has been shown to modulate growth factor and cytokine signaling and to hold tumor suppressor activity in breast, pancreas, kidney, and hepatocellular cancer cell lines [41]. Moreover, it has been reported that SULF1is downregulated in PC, where it has been suggested to inhibit growth of PC bone metastases [42]. The role of TULP4, MKLN1, and ZNF532 in cancer is largely unknown.

Lastly, we were able to provide proof-of-principle that a subset of our circRNA candidates can be detected also in minimally invasive EV-enriched plasma samples from PC patients. Interestingly, we found that the overall level of circRNAs in EV-enriched plasma was significantly higher in samples from MPC patients compared to samples from cancer-free controls (*P* = 0.03, Fig. 4h), in contrast to the global downregulation observed in PC compared to AN tissue samples (*P* < 0.0001, Fig. 2f). While circRNAs have been comprehensive profiled in both serum [12] and extracellular vesicles from cancer cell lines (liver, colon, lung, stomach, breast, and cervical cancers) [13], the former studies were limited by using very few patient serum samples (*n* = 2) or cell lines. However, few other studies have previously detected circRNAs in plasma/serum samples from PC patients [43–48], the majority of which focused on a single or two selected circRNAs, which is most likely the reason for the lack of overlap between the previously reported circRNAs detected in serum/plasma and the 39 circRNA candidates in this study.

However, similar to our findings, four of the previous studies reported an elevation of the respective circRNA investigated between PC patients and controls [44–46, 48]. Further, we found that circACVR2A levels were elevated in EV-enriched plasma samples from LPC patients with high *vs.* low GG tumors (*P* = 0.10, Fig. 4l), similar to a previous report of elevated circAR3 in plasma samples from PC patients with high GG tumors [45]. Combined, these results suggest a possible association between circRNA levels in EV-enriched plasma and tumor burden, supporting the potential of circRNAs as minimally invasive biomarkers in PC. However, further studies, including larger cohorts, are needed to fully assess the clinical utility.

The PC patient cohorts 1–3 had some different characteristics. Clinical follow-up for cohort 2 was short and incomplete (median 20 months), thereby preventing evaluation of prognostic biomarker potential. Clinical follow-up time for RP cohort 1 was 66 months, whereas it was 125 months in RP cohort 3, thus allowing for analysis of progression to MPC as an additional endpoint besides BCR in RP cohort 3.

Future studies should also evaluate the prognostic potential of the 5-circRNA signature in diagnostic biopsies to assess if the 5-circRNA signature can improve risk stratification and initial treatment decisions at time of diagnosis.

Furthermore, circRNA annotation can be biased based on the algorithm used, and it has been reported that multiple algorithms may be used to ensure reliable predictions from RNA-seq data [49]. Still, we confirmed the presence of our 39 top candidate circRNAs, identified by the CIRI2 pipeline in RNA-seq data in cohort 1, using an independent method (NanoString) as well as two independent patient cohorts (cohorts 2 and 3), supporting the validity of our findings.

In conclusion, our results highlight a profound and robust dysregulation of circRNAs between PC and AN samples, and importantly, illustrate a strong prognostic biomarker potential for our 5-circRNA signature across two independent RP cohorts. If successfully validated in future studies, the 5-circRNA signature could aid in selecting patients with a high probability of BCR post-surgery for intensified treatment following RP. Additional investigations into the use of circRNAs as minimally invasive biomarkers in PC are encouraged by our promising findings of specific circRNAs being significantly elevated in EV-enriched plasma from MPC patients compared to cancer-free controls.

## Conclusions

Using tissue and plasma samples from four distinct patient cohorts, we have provided the largest and most comprehensive circRNA profiling study of PC to date. We found that circRNAs hold great biomarker potential in PC, with several circRNAs being highly cancer specific and/or associated with disease progression. Further studies, including larger cohorts, are required to fully assess the clinical utility of circRNAs in future PC management.

## Supplementary Information

**Additional file 1.** Supplementary Methods.

**Additional file 2.** Fig. S1: Flow charts of inclusion/exclusion in cohort 1 (training) according to the REMARK guidelines. Fig. S2: Flow charts of inclusion/exclusion for cohorts 2-4. Fig. S3: Clustering of abundant circRNAs in cohort 1. Fig. S4: Association of abundant circRNAs to metastatic disease. Fig. S5: Association of abundant circRNAs to key clinicopathological parameters. Fig. S6: circKDM1A holds prognostic potential in prostate cancer patients. Fig. S7: circRNA candidates show both correlations and substantial differences between levels in EV-enriched plasma and in prostate tissue samples.

**Additional file 3.** circRNA top biomarker candidates selected for validation.

**Additional file 4.** Dysregulated circRNAs in cohort 1.

**Additional file 5.** Association of abundant circRNAs to clinicopathological parameters in RP cohort 1.

**Additional file 6.** Validation of dysregulated circRNA top candidates.

**Additional file 7.** Validation of circRNA candidates associated with clinicopathological parameters.

**Additional file 8.** Uni- and multivariate Cox regression analyses of biochemical recurrence-free survival time in RP cohorts 1 and 3.

**Additional file 9.** Detectability of circRNA top candidates in EV-enriched plasma samples.

**Additional file 10.** Expression data of circRNA in cohort 1.

**Additional file 11.** Expression data of circRNA in cohort 2.

**Additional file 12.** Expression data of circRNA in cohort 3.

**Additional file 13.** Expression data of circRNA in cohort 4.

## Data Availability

The circRNA expression data from cohorts 1–4 are provided in Additional files 10-13, Tables S8-11. As the requirement for patient consent was waived in the current study, we do not have permission to deposit the full raw and individual level clinical data in a (controlled access) repository. Instead, raw data and individual level clinical data is available from the corresponding author upon reasonable request due to Danish data protection laws. Under Danish law, access to this data will require: 1) Ethical approval of the new research project, which has to be applied for to the relevant Danish authority by the data owner (corresponding author of this work, Aarhus University Hospital) on behalf of the PI/collaborator of the new project. 2) After ethical approval has been obtained, a data sharing agreement for the new project is required between the data owner (corresponding author, Aarhus University Hospital) and the PI/collaborator of the project and their institution(s).

## Abbreviations

ADT: Androgen deprivation therapy
AN: Adjacent-normal
AUC: Area under the curve
BCR: Biochemical recurrence
BH: Benjamini-Hochberg
bp: Base pairs
BSJ: Back-splice junction
CI: Confidence interval
C-index: Harrell’s Concordance Index
circRNAs: Circular RNAs
CRC: Colorectal cancer
EV: Extracellular vesicle
FC: Fold change
FF: Fresh-frozen
FFPE: Formalin-fixed paraffin-embedded
GG: Gleason Grade Group
HR: Hazard ratio
LPC: Localized prostate cancer
MPC: Metastatic prostate cancer
NMF: Non-Negative Matrix Factorization
PC: Prostate cancer
PSA: Prostate-specific antigen
ROC: Receiver operating characteristics
RP: Radical prostatectomy
TRUP: Transurethral Resection of The Prostate
TRUSbx: Transrectal ultrasound-guided biopsy
