# Supplementary material for: The transcriptional landscape and biomarker potential of circular RNAs in prostate cancer
Source: Genome Med. 2022 Jan 25;14:8. doi: 10.1186/s13073-021-01009-3 (PMC8788096; doi:10.1186/s13073-021-01009-3)
Supplement: Supplementary file 1 — Additional file 1. Supplementary Methods. [file 13073_2021_1009_MOESM1_ESM.docx]

**The Transcriptional Landscape and Biomarker Potential of circular RNAs in Prostate Cancer**

Emma Bollmann Hansen, Jacob Fredsøe, Trine Line Hauge Okholm, Benedicte Parm Ulhøi, Søren Klingenberg, Jørgen Bjerggaard Jensen, Jørgen Kjems, Kirsten Bouchelouche, Michael Borre, Christian Kroun Damgaard, Jakob Skou Pedersen, Lasse Sommer Kristensen, Karina Dalsgaard Sørensen^*^

***Corresponding author**:

Karina Dalsgaard Sørensen, Professor, MSc, PhD

Department of Molecular Medicine (MOMA), Aarhus University Hospital (AUH) & Department of Clinical Medicine, Aarhus University (AU)

Palle Juul-Jensens Boulevard 99, 8200 Aarhus N, Denmark

Tel: (+45) 7845 5316

Email: [kdso@clin.au.dk](mailto:kdso@clin.au.dk)

# Supplementary Methods

## Patient cohorts

**Cohort 1 - total RNA-seq profiling of circRNAs:**

Cohort 1 included 31 adjacent-normal (AN) and 126 tumor samples from 141 patients with clinically localized prostate cancer (LPC), as well as 17 primary tumor samples from metastatic prostate cancer (MPC) patients.

*RP cohort 1* (subset of cohort 1) included 126 curatively intended radical prostatectomy (RP) samples of histologically verified clinically LPC from the Department of Urology, Aarhus University Hospital (2004-2017). Patients in RP cohort 1 were included if they had at least two years of follow up available.

Immediately following RP, fresh prostate tissue biopsies were obtained and stored at -80°C in TissueTek. Prior to RNA extraction, TissueTek blocks were sliced in half and the first half of each TissueTek block were cut in approximately 40 sections (20 µm thickness). The first and last tissue sections from each half block were stained with Hematoxylin and Eosin (HE) and evaluated by a trained pathologist to assess areas of prostate cancer tissue. Total RNA from macrodissected tissue was purified with RNeasy Plus Mini Kit (QIAGEN, Cat#74036), according to the manufacturer’s description. RNA concentration was measured on NanoQuant Plate^TM^ (TECAN), and RNA integrity was measured using the 2100 Bioanalyzer (Agilent), on a Nano or Pico chip dependent on the size of the macrodissected tissue area. Of RNA samples used for total RNA-seq, 92% had ≥ 400ng RNA and RIN ≥ 7.

Initially, 219 patients meeting the inclusion criteria for RP cohort 1, had available tumor tissue in retrieved TissueTek blocks. Of these 219 patients, one was excluded due to pre- or postoperative adjuvant radiation or endocrine therapy. Of the remaining 218 patients, 92 were excluded because of poor tissue quality (n=10) or poor RNA quality/different tissue histology in the first and last tissue sections (n=82), leaving 126 samples in total in RP cohort 1 (Suppl. Fig. S1a).

*AN samples (cohort 1):* Fresh frozen (FF) AN tissue specimens were sampled from RPs of histologically verified clinically LPC from the Department of Urology, Aarhus University Hospital (2004-2017). For patients with cancer in only one of the prostate lobes (left/right), the opposing lobe were examined for AN tissue. TissueTek blocks were evaluated and RNA extracted as described for RP cohort 1 (Suppl. Fig. S1c).

*MPC samples (cohort 1)*: FF primary tumor specimens from MPC patients were obtained from patients undergoing palliative transurethral resection of the prostate (TURP) at the Department of Urology, Aarhus University Hospital (2007-2008) or the Department of Urology, Regional Hospital West Jutland (2016-2019). TissueTek blocks were evaluated and RNA extracted as described for RP cohort 1 (Suppl. Fig. S1b).

**Cohort 2 – NanoString validation of circRNA candidates:**

Cohort 2 consisted of Formalin-Fixed Paraffin-Embedded (FFPE) samples of 22 AN samples, 35 tumor samples from clinically LPC patients, and 54 primary tumor samples from MPC patients.

LPC samples were sampled from RP specimens (n=22) or diagnostic transrectal ultrasound guided needle biopsies (TRUSbx) (n=14), MPC samples were sampled from diagnostic TRUSbx, and AN samples were sampled from RP specimens (n=18) or diagnostic TRUSbx (n=4). All samples were obtained from the Department of Pathology, Aarhus University Hospital, Denmark, or the Department of Pathology, Viborg Regional Hospital, Denmark (collected from 2017 to 2019).

All patients in cohort 2 have been previously described in [1]. We selected 116 patients meeting initial inclusions criteria: FFPE tissue sampled with a fixation age <3.0 years, a clear PSMA-positive primary tumor burden evaluated on the ^68^Ga-PSMA PET/CT, no multifocal tumors in the prostate evaluated on the ^68^Ga-PSMA PET/CT, and no earlier malignancies except benign skin cancer.

Of these 116 patients, 6 patients had missing FFPE blocks, leaving 110 patients with 155 samples available (45 AN, 41 LPC, 69 MPC). Of these 155 patient samples, 41 samples had too poor RNA quality (21 AN, 5, LPC, and 15 MPC). Three samples failed in NanoString analysis (2 AN and 1 LPC), leaving 111 samples eligible for the final analysis (22 AN, 35 LPC, 54 MPC, cohort 2, Suppl. Fig. S2a).

Prior to RNA extraction, a trained pathologist evaluated all tissue specimens. After pathological grading using the 2014 ISUP system (Gleason Grade Group), punch biopsies (1.5 mm) of representative areas of marked tumor were taken from the corresponding paraffin block, as previously described [2]. Total RNA was isolated from the punch biopsies using the miRNeasy FFPE Kit (QIAGEN, Cat#217504), according to the protocol provided by the manufacturer. Samples with low quality RNA (260/280 ratio <1.75 or <300ng RNA) were excluded from NanoString analysis.

**RP Cohorts 3 – NanoString validation of circRNA candidates:**

RP cohort 3 consisted of 191 curatively intended RPs of histologically verified clinically LPC from the Department of Urology, Aarhus University Hospital (1998-2009). Patients were excluded if they had received either pre- or postoperative adjuvant radiation or endocrine therapy.

For RP cohort 3, 461 patients from an earlier study were selected ([3], cohorts 1 and 2). Of these 461 patients, 215 had samples with enough RNA left (>300 ng RNA) for NanoString analysis. In NanoString analysis, 17 samples failed, leaving 191 samples eligible for the final analysis (RP cohort 3, Suppl. Fig. S2b). FFPE samples from RP specimens were evaluated, biopsied, and RNA extracted as described for cohort 2.

**Cohort 4 – NanoString profiling of circRNA candidates in liquid biopsies:**

Cohort 4 consisted of 48 men undergoing initial TRUSbx (10-12 needles) due to suspicion of prostate cancer and 6 patients with known MPC undergoing palliative TURP. Of the 48 patients undergoing initial TRUSbx, 27 patients were histologically verified as cancer-free in all needles (controls), while 21 patients were diagnosed with clinically LPC. Plasma samples from controls and LPC patients were collected at the Department of Urology, Aarhus University Hospital, Denmark (2017-2020). Plasma samples from MPC patients were collected at Department of Urology, Regional Hospital West Jutland, Denmark (2016-2019). All patients with clinically LPC patients included in the cohort subsequently underwent curatively intended RP for histologically verified clinically LPC at the Department of Urology, Aarhus University Hospital, Aarhus, Denmark (2017-2020). Blood samples were drawn into tubes containing EDTA just prior to TRUSbx or TURP. In all cases, whole blood was spun at 2000x *g* for 10 min at 4°C before the plasma was transferred into fresh cryotubes and stored at -80°C until use.

An enrichment of extracellular vesicles (EVs) was performed using miRCURY Exosome Serum/Plasma Kit (QIAGEN, Cat#76603) from 1.4 µl plasma, according to the manufacturer’s protocol. Total RNA was purified using miRNeasy Micro Kit (QIAGEN, Cat#217084), eluated in 14µL RNase-free water, and stored at -80°C until NanoString analysis using the same custom panel used for validation in tissue samples.

Patients and controls were included if they had a plasma sample collected at time of initial TRUS or TURP and if the plasma sample was no more than five years old.

A total of 60 plasma samples were analyzed using the custom NanoString nCounter codeset (31 control, 23 LPC, 6 MPC). Of these 60 samples, 6 failed NanoString analysis (4 control and 2 LPC), leaving 54 eligible for the final analysis (27 control, 21 LPC, 6 MPC, Suppl. Fig. S2c).

**References to Supplementary Methods**

1. Klingenberg, S., M.R. Jochumsen, B.P. Ulhøi, J. Fredsøe, K.D. Sørensen, M. Borre, et al., *(68)Ga-PSMA PET/CT for Primary Lymph Node and Distant Metastasis NM Staging of High-Risk Prostate Cancer.* J Nucl Med, 2021. **62**(2): p. 214-220.

2. Haldrup, C., K. Mundbjerg, E.M. Vestergaard, P. Lamy, P. Wild, W.A. Schulz, et al., *DNA Methylation Signatures for Prediction of Biochemical Recurrence After Radical Prostatectomy of Clinically Localized Prostate Cancer.* Journal of Clinical Oncology, 2013. **31**(26): p. 3250-3258.

3. Laursen, E.B., J. Fredsøe, L. Schmidt, S.H. Strand, H. Kristensen, A.K.I. Rasmussen, et al., *Elevated miR-615-3p Expression Predicts Adverse Clinical Outcome and Promotes Proliferation and Migration of Prostate Cancer Cells.* The American Journal of Pathology, 2019. **189**(12): p. 2377-2388.
