# Supplementary material for: The transcriptional landscape and biomarker potential of circular RNAs in prostate cancer
Source: Genome Med. 2022 Jan 25;14:8. doi: 10.1186/s13073-021-01009-3 (PMC8788096; doi:10.1186/s13073-021-01009-3)
Supplement: Supplementary file 2 — Additional file 2. Fig. S1: Flow charts of inclusion/exclusion in cohort 1 (training) according to the REMARK guidelines. Fig. S2: Flow charts of inclusion/exclusion for cohorts 2-4. Fig. S3: Clustering of abundant circRNAs in cohort 1. Fig. S4: Association of abundant circRNAs to metastatic disease. Fig. S5: Association of abundant circRNAs to key clinicopathological parameters. Fig. S6: circKDM1A holds prognostic potential in prostate cancer patients. Fig. S7: circRNA candidates show both correlations and substantial differences between levels in EV-enriched plasma and in prostate tissue samples. [file 13073_2021_1009_MOESM2_ESM.docx]

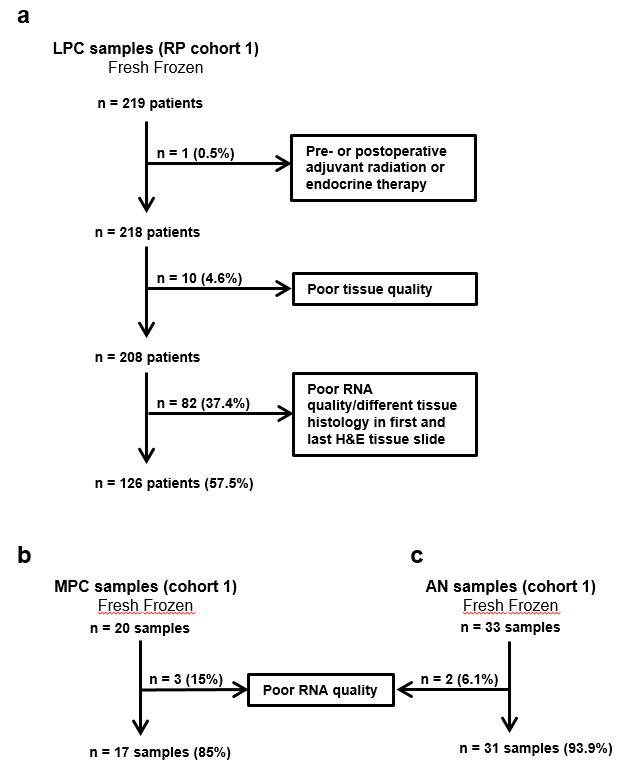


**Fig. S1: Flow charts of inclusion/exclusion in cohort 1 (training) according to the REMARK guidelines. a**: RP cohort 1; **b**: MPC samples; **c**: AN samples.

**
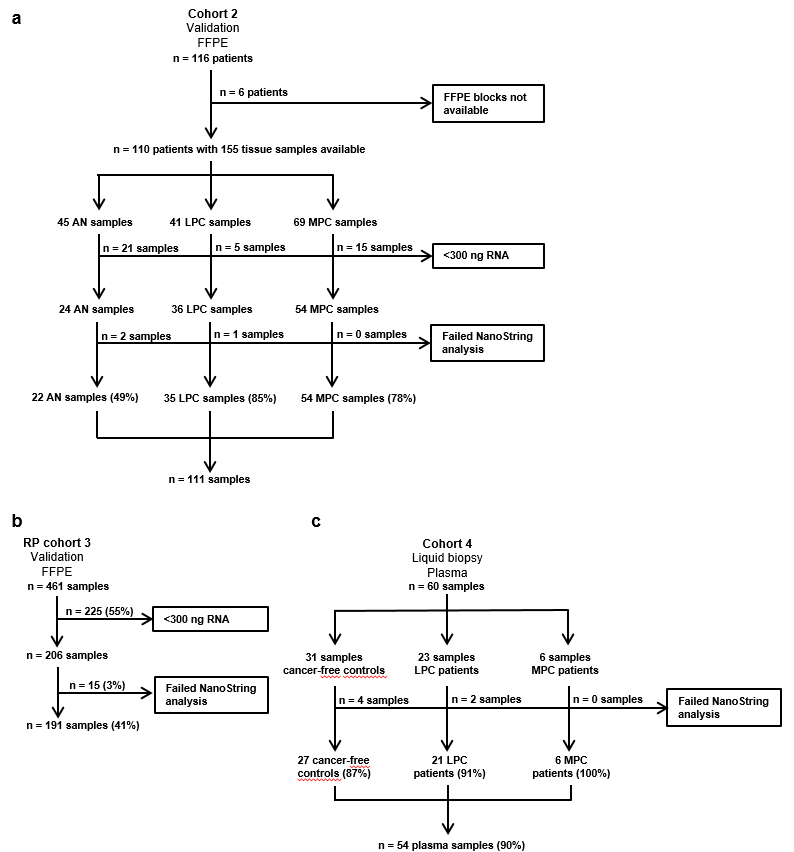
**

**Fig. S2: Flow charts of inclusion/exclusion for cohorts 2-4. a**: cohort 2; **b**: RP cohort 3; **c**: cohort 4.


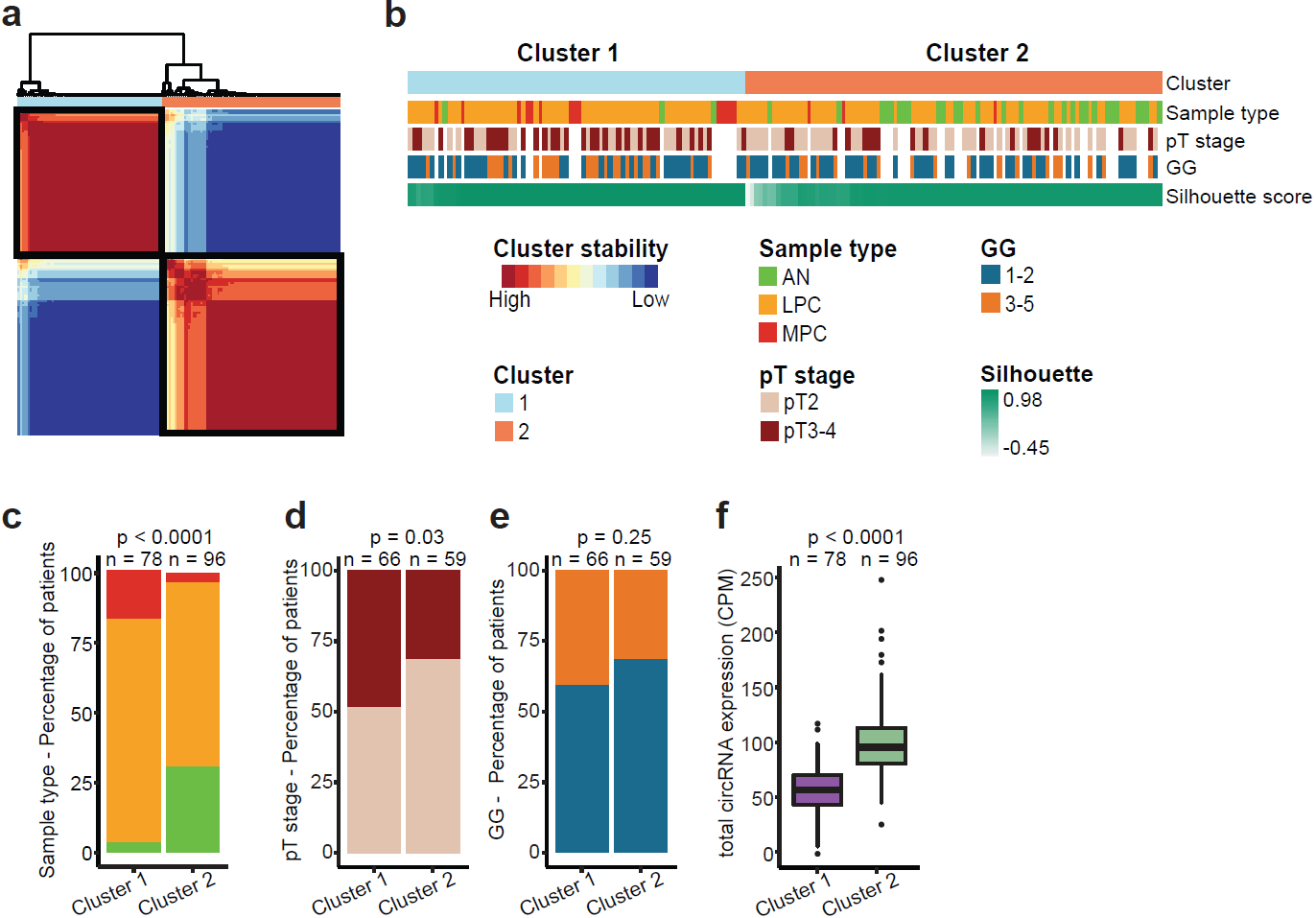


**Fig. S3: Clustering of abundant circRNAs in cohort 1. a:** Consensus matrix for two clusters based on the 100 most variable of the abundant circRNAs in cohort 1. Samples are in both rows and columns and pairwise values range from dark blue (samples never cluster together) to dark red (samples always cluster together). **b:** Sample types and clinicopathological information for samples stratified by circRNA expression. Samples are ordered after increasing silhouette score within each cluster. **c-f:** circRNA clusters compared to **c:** sample type (AN, LPC, MPC), **b:** pT stage, **c:** GG, and **f**: total circRNA expression.


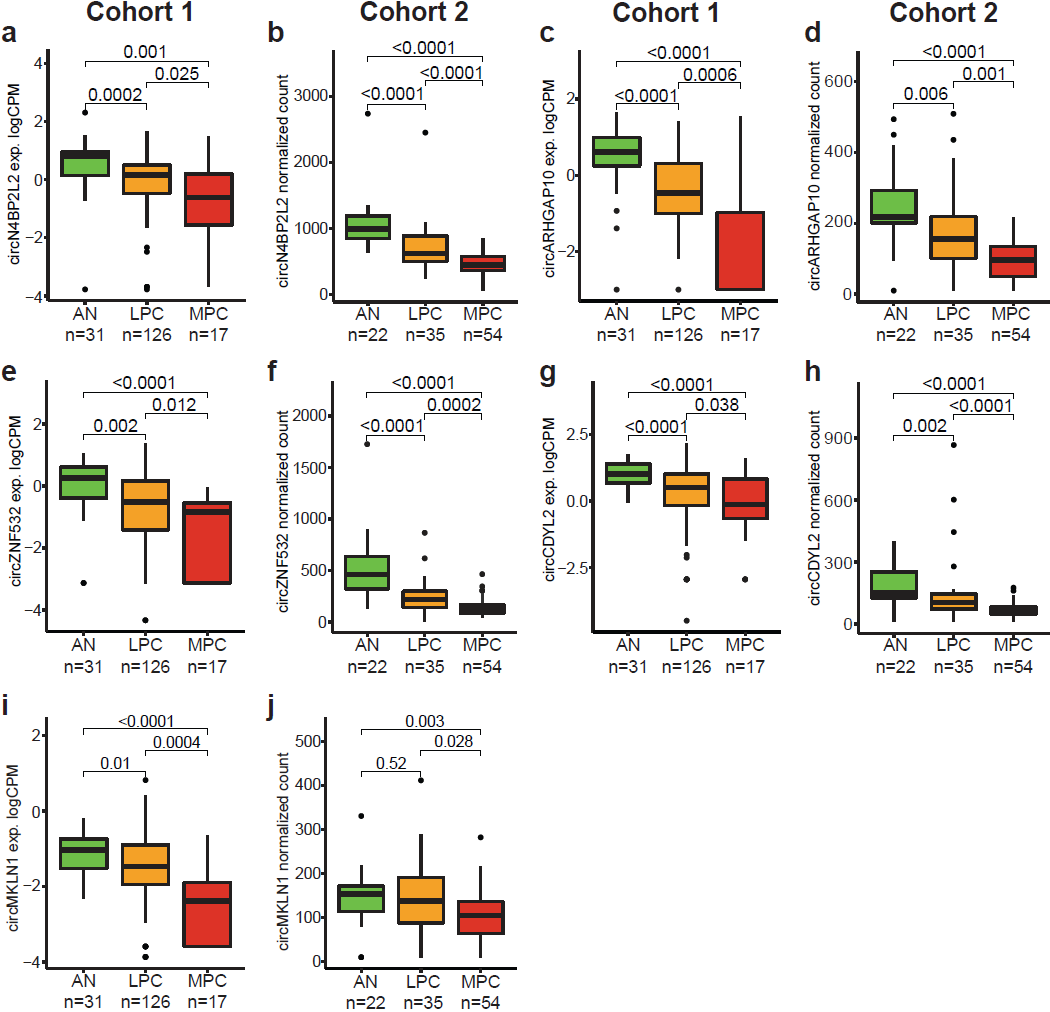


**Fig. S4: Association of abundant circRNAs to metastatic disease.** Boxplot of individual circRNA expression compared to sample types (AN, LPC, MPC; **a, c, e, g, i**: cohort 1; **b, d, f, h, j**: cohort 2). P values represent Wilcoxon rank-sum test. Boxes represent the 25^th^ and 75^th^ percentiles and median. Outlier cases, defined as more than 1.5 times the IQR from the median are marked as individual dots outside the whiskers.


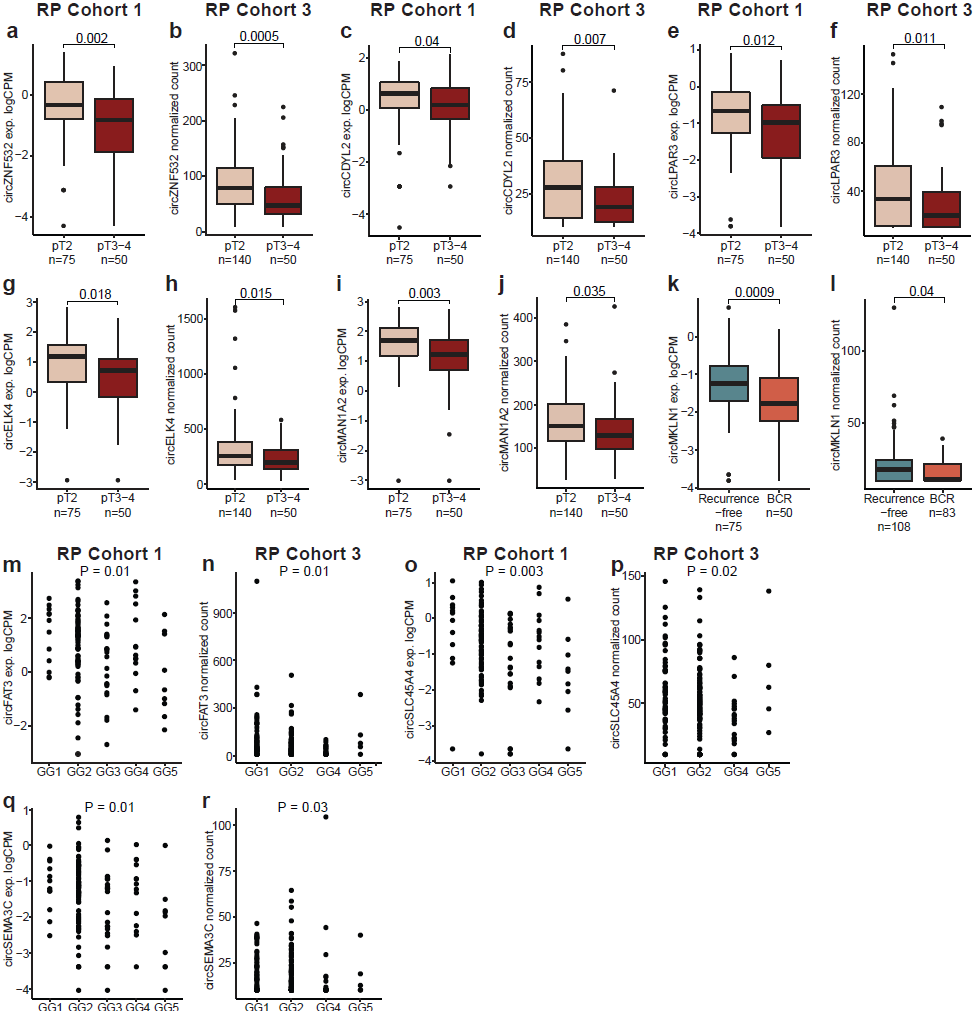


**Fig. S5: Association of abundant circRNAs to key clinicopathological parameters.** Boxplot of individual circRNA expression compared to **a-j:** pT stage, **k-l:** BCR status (**a, c, e, g, i, k**: RP cohort 1; **b, d, f, h, j, l**: RP cohort 3). P values represent Wilcoxon rank-sum test. Boxes represent the 25^th^ and 75^th^ percentiles and median. Outlier cases, defined as more than 1.5 times the IQR from the median are marked as individual dots outside the whiskers. **m-r:** scatter plot of GG (**m, o, q**: RP cohort 1; **n,p,r**: RP cohort 3). P values represent Kendall's rank correlation.


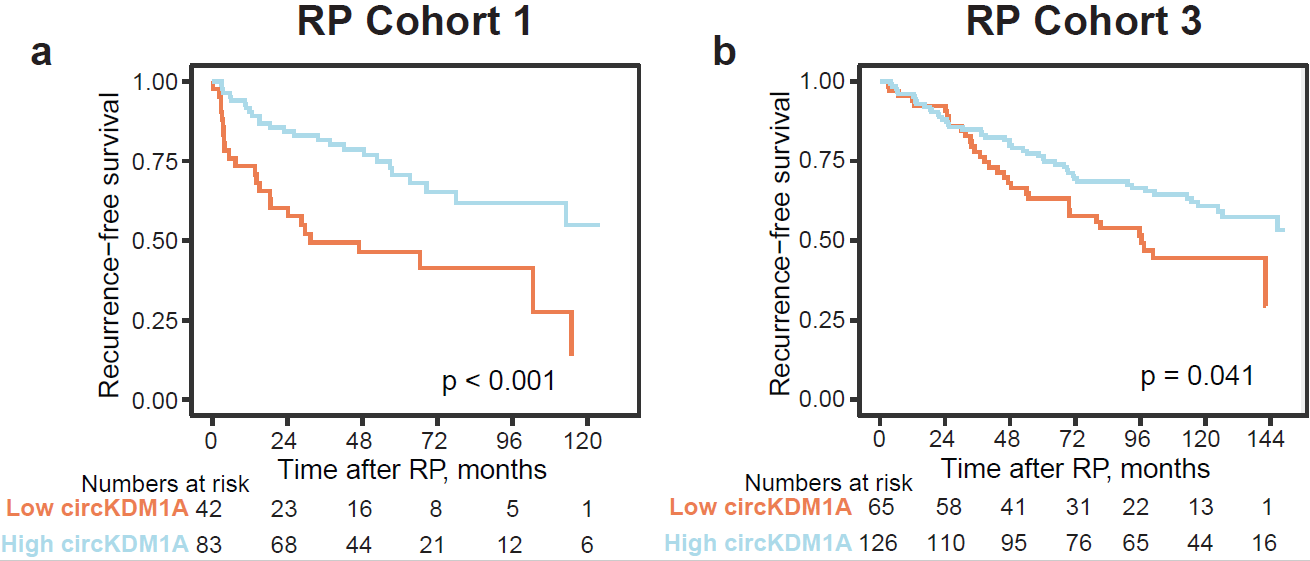


**Fig. S6: circKDM1A holds prognostic potential in prostate cancer patients.** Kaplan Meier analysis of biochemical recurrence (BCR)-free survival in RP cohort 1 (**a**) and RP cohort 3 (**b**). Patients in RP cohorts 1 and 3 were dichotomized based on cut-off trained in RP cohort 1 from circKDM1A expression. For each Kaplan Meier plot, p values for two-sided log-rank tests and the number of patients at risk are given.


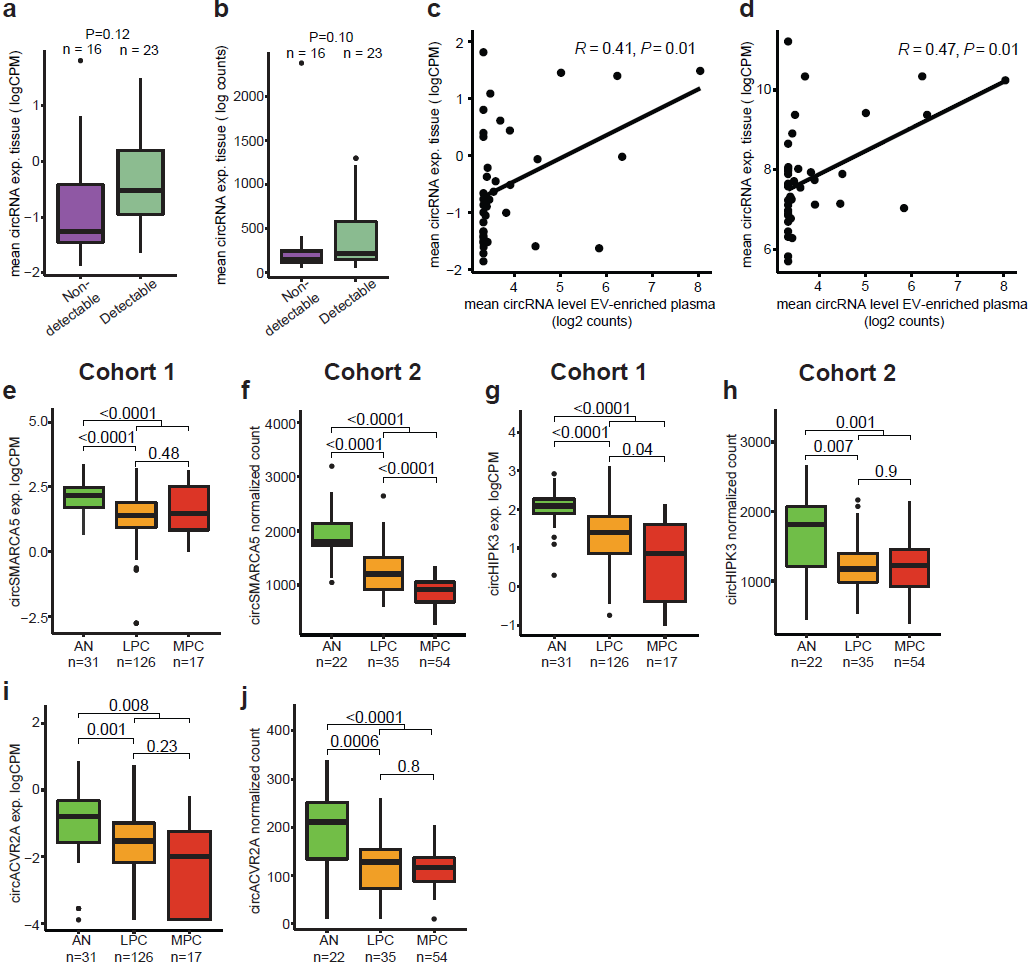


**Fig. S7: circRNA candidates show both correlations and substantial differences between levels in EV-enriched plasma and in** **prostate tissue samples. a-b:** Boxplot of mean circRNA candidate expression in tissue samples from cohort 1 (**a**) and cohort 2 (**b**) compared to the detectability of the specific circRNA in EV-enriched plasma from patients in cohort 4 (n=54). P values represent Wilcoxon rank-sum test. **c-d**: For each circRNA candidate, the mean expression in tissue samples from cohort 1 (**c**) or cohort 2 (**d**) *vs.* mean level in EV-enriched plasma from patients in cohort 4. P values represent Kendall's rank correlation. **e-j:** Boxplot of circRNA expression in tissue samples compared to sample type (AN, LPC, MPC; **e, g, i**: cohort 1; f**, h, j**: cohort 2). P values represent Wilcoxon rank-sum test. Boxes represent the 25^th^ and 75^th^ percentiles and median. Outlier cases, defined as more than 1.5 times the IQR from the median are marked as individual dots outside the whiskers.
